# Exacerbation of pleural effusion in a dasatinib-treated patient during the perioperative period

**DOI:** 10.1186/s40981-022-00577-6

**Published:** 2022-10-24

**Authors:** Takayuki Hasegawa, Satoki Inoue, Keisuke Yoshida, Masahiko Akatsu

**Affiliations:** 1 Department of Anesthesiology, Fukushima Medical University, 1 Hikarigaoka, Fukushima, Fukushima 960-1295 Japan; 2 Department of Anesthesiology, Iwaki City Medical Center, 16 Kusehara, Uchigomimayamachi, Iwaki, Fukushima 973-8555 Japan

**Keywords:** Dasatinib, Pleural effusion, Ultrasonography

To the editor,

Dasatinib is one of the first-line drugs for chronic myeloid leukemia in chronic phase (CML-CP) [1]. Dasatinib-related pleural effusion has been reported in 28–33% of patients taking dasatinib and severe pleural effusion in 3–7.3% [2]. We herein report a case of perioperative dasatinib-related severe pleural effusion.

A 57-year-old Japanese man (body weight, 67 kg; height, 166 cm) with a history of CML-CP was admitted for surgical removal of a dentigerous cyst of the maxilla. He had been taking dasatinib 100 mg once a day for 3 years without side effects. Preoperative examinations showed no abnormal findings except for a small left pleural effusion on chest radiograph performed 27 days before surgery (Fig. 1a).

**Fig. 1:**
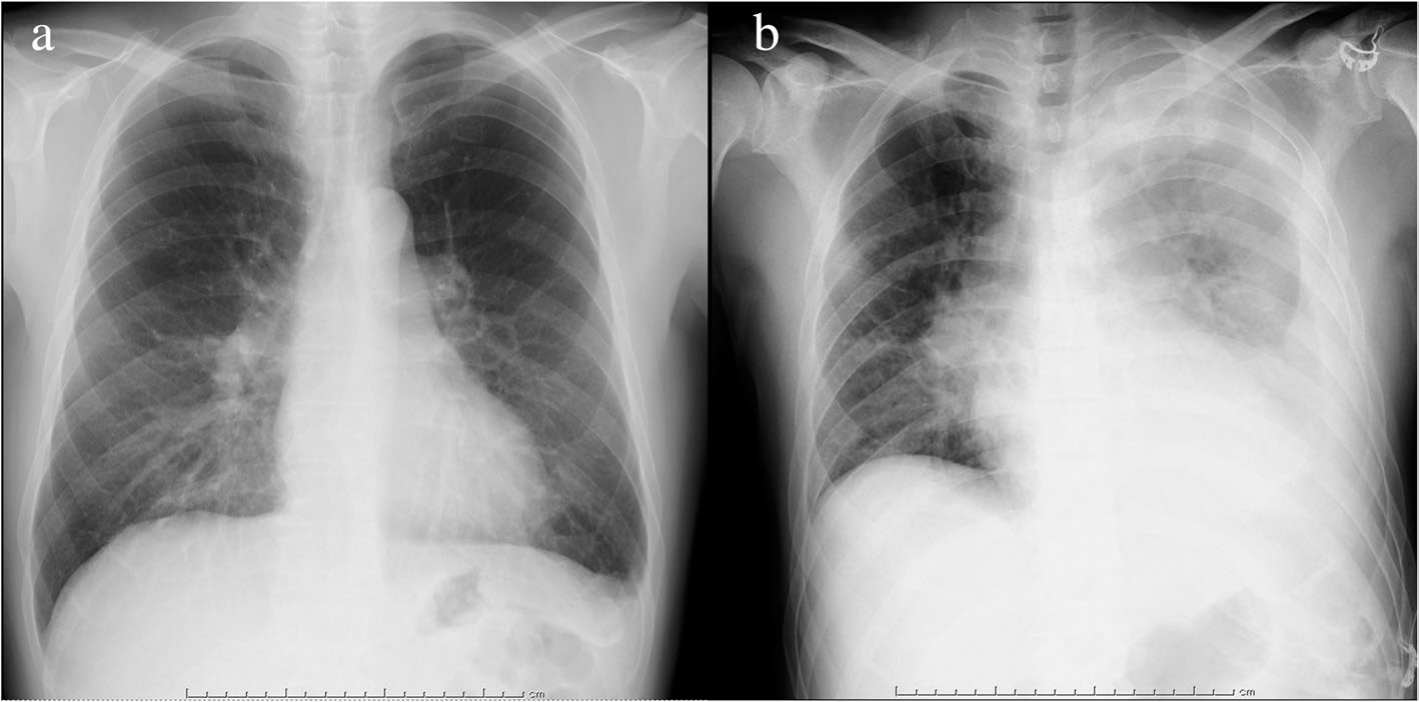
**a** Chest radiograph image taken 27 days before surgery, showing a small left pleural effusion. **b** Chest radiograph image taken immediately following surgery, showing a large volume of left pleural effusion

The patient had no respiratory symptoms during daily activities such as stair climbing. The respiratory rate and SpO2 were 20/min and 94% on room air, respectively, immediately before the patient was brought into the operating room, and again immediately before general anesthesia was induced, after which nasotracheal intubation was performed. Post-intubation auscultation was routinely performed in the ventral lung field, but no abnormal findings were detected. Desflurane was used for the maintenance of anesthesia. Intraoperatively, FiO_2_ was set to 0.5, tidal volume to 450 mL, and positive end-expiratory pressure to 5 cm H_2_O; SpO_2_ remained at 94–95% and peak airway pressure was 15 cm H_2_O. The surgery was completed in 49 min without any trouble. Sugammadex 200 mg was administered after four twitches to train-of-four stimulation appeared. After that, the patient could respond to verbal commands, his spontaneous respiratory rate was 12/min and his trachea was extubated. After 3 min, he complained of dyspnea, and his SpO_2_ decreased to 90% while he was receiving 10 L/min of oxygen. A chest radiograph taken in the operating room revealed a large volume of left pleural effusion (Fig. 1b). His trachea was reintubated, thoracentesis was performed, and 1100 mL of light-yellow exudative fluid was drained. Cytology showed lymphocyte predominance (98%), which is a characteristic feature of dasatinib-induced pleural effusion [3]. Mechanical ventilation was continued for 2 days, pleural effusion was decreased, and his trachea was extubated without complications. On the 16th postoperative day, the pleural effusion had disappeared, and dasatinib was resumed. However, the pleural effusion developed and increased in size, and dasatinib was stopped 51 days after resumption.

It is possible that because the increase in pleural effusion was gradual, the patient’s tolerance was acceptable until anesthesia induction which, however, caused a breakdown in tolerance and a collapse in respiratory status. Indeed, it is possible that the pleural effusion increased rapidly only during anesthesia; however, this is unlikely because the effusion did not increase postoperatively. We were able to draw the following from the current case. First, perioperative patients treated with dasatinib are typically at risk for respiratory complications, even in those with no history of respiratory disorders. Risk factors for dasatinib-related pleural effusion include a history of cardiac disease, hypertension, hypercholesterolemia, autoimmune disease, or skin rash during or prior to dasatinib treatment, as well as a twice-daily dasatinib schedule [3, 4]. However, there have been no reports on risk factors related to the severity of dasatinib-related pleural effusion. We considered that the most plausible cause of pleural effusion in our patient was dasatinib. Second, ultrasonography should be more readily available perioperatively. Almost all anesthesiologists would be somewhat concerned if their patient’s SpO_2_ was 94% before induction of anesthesia. It would have been better to prioritize examinations over execution of anesthesia. If we had performed ultrasonography, which is easier to perform than other examinations, its findings may have contributed to the detection of increased pleural effusion and prevention of deterioration in the patient’s condition.

## Data Availability

Data sharing is not applicable to this article, as no datasets were generated or analyzed for the report.

## Abbreviations

CML-CP: Chronic myeloid leukemia in chronic phase
SpO_2_: Peripheral capillary oxygen saturation
FiO_2_: Fraction of inspired oxygen
